# HIV Among Indigenous peoples: A Review of the Literature on HIV-Related Behaviour Since the Beginning of the Epidemic

**DOI:** 10.1007/s10461-015-1023-0

**Published:** 2015-03-03

**Authors:** Joel Negin, Clive Aspin, Thomas Gadsden, Charlotte Reading

**Affiliations:** 1 Sydney School of Public Health, University of Sydney, Edward Ford Building (A27), Sydney, NSW 2006 Australia; 2 Independent consultant, Sydney, Australia; 3 School of Public Health and Social Policy, Faculty of Human and Social Development, University of Victoria, Victoria, Canada

**Keywords:** Indigenous peoples, HIV, Colonization

## Abstract

From the early days of the HIV epidemic, Indigenous peoples were identified as a population group that experiences social and economic determinants—including colonialism and racism—that increase exposure to HIV. There are now substantial disparities in HIV rates between Indigenous and non-Indigenous peoples in some countries. We conducted a comprehensive literature review to assess the evidence on HIV-related behaviors and determinants in four countries—Australia, Canada, New Zealand and the United States—in which Indigenous peoples share important features of colonization and marginalization. We identified 107 articles over more than 20 years. The review highlights the determinants of HIV-related behaviors including domestic violence, stigma and discrimination, and injecting drug use. Many of the factors associated with HIV risk also contribute to mistrust of health services, which in turn contributes to poor HIV and health outcomes among Indigenous peoples.

## Introduction

Across the globe, the impact of HIV has been particularly pernicious among socially and economically marginalized groups, such as men who have sex with men (MSM), sex workers and people who inject drugs (PWID). From the early days of the HIV epidemic, Indigenous peoples in some countries were identified as a population group that also experiences social and economic determinants that increase exposure to HIV [1, 2]. However, the official response to this vulnerability has often been limited and ineffectual. As well, relatively little has been written about what is driving rising rates of HIV among Indigenous peoples and, more than three decades after HIV was first detected, it is clear that HIV has become a critical health issue for these historically oppressed populations [3].

Indigenous peoples have long-standing connections to their ancestral lands, dating back many generations and pre-dating colonization. Although cultural, linguistic, and geographic differences exist within and across Indigenous populations globally, to a large extent, colonialism, racism, social exclusion, and the repression of self-determination act as the determinants within which Indigenous health is constructed [4, 5].

The past decade has seen considerable emphasis on and recognition of the social determinants of health: the economic and social conditions that influence differences in health status. These include social exclusion, experiences in early life, education, stress, social support, employment factors, housing, addiction, food and transport. Marmot and colleagues work in this area has highlighted the vast individual and group level differences in health outcomes driven by disparities across these various determinants [6–8]—factors that are particularly relevant to Indigenous peoples.

In some countries, research now clearly indicates a link between the distinct social inequities experienced by Indigenous peoples and higher rates of HIV than non-Indigenous peoples [9]. Similarly, disparities in the timing of diagnoses as well as treatment outcomes between Indigenous and non-Indigenous peoples in the same country pose significant challenges for health services and government agencies as we enter the fourth decade of the HIV epidemic.

This study focuses on four countries—Australia, New Zealand, Canada and the United States—where Indigenous peoples represent a statistical minority and where Indigenous peoples share a history of political and social marginalization that extends into health access and outcomes. We acknowledge that there are Indigenous peoples beyond these four countries who also experience similar aspects of poverty and alienation. However, our focus on these four countries was due largely to the common experience of colonization, displacement and neglect, which have contributed to health and social disparities between Indigenous and non-Indigenous peoples. In these countries, Indigenous peoples have higher mortality and morbidity as well as poorer health outcomes than their non-Indigenous peers [10, 11]. Colonization saw a dislocation from traditional lands and cultures among Indigenous peoples who had and continue to have strong links to land. This alienation from territory and domination by outsiders adversely affected physical, social and emotional wellbeing by suppressing traditional life and customs [12, 13]. Subsequent legal, political and social marginalization was often coupled with racial prejudice to produce a reality of poverty, underemployment and poor education [14]. Residential schools and “stolen generations” exacerbated displacement and loss of identity and were often associated with physical and sexual abuse that affected future generations [15, 16].

Table 1 presents contextual data on demographic characteristics and HIV prevalence between Indigenous and non-Indigenous peoples in the four study countries. Rates of poverty among Indigenous peoples in these countries are higher than among non-Indigenous people. For example, in the United States, about 28 % of Indigenous peoples lived in poverty in 2000 compared to 12 % of the total population [17].

**Table 1:** Demographic characteristics and HIV prevalence in four study countries [3]

Country	Population	Indigenous population	Indigenous population as percentage of total (%)	HIV diagnosis rates (per 100,000) Indigenous peoples	HIV diagnosis rates (per 100,000) non-Indigenous people
Australia	21,507,719 (2011)	548,370 (2011)	2.5	31.2	26.2
Canada	32,852,320 (2011)	1,400,685 (2011)	4.3	179.2	29.2
New Zealand	4,433,100 (2012)	682,200 (2012)	15.4	18.9	18.5
United States	308,745,538 (2010)	5,220,579 (2010)	1.7	9.3	7.0 (White only)

United States HIV diagnosis rate information from Centers for Disease Control and Prevention [21]. Population data from most recent census [22] and from Statistics New Zealand [23]. Overall rates for Canada, Australia and New Zealand are standardized directly to the age distribution of the 2001 Canadian male Indigenous population. HIV diagnoses from national surveillance and notification systems

Among Indigenous peoples in Canada, HIV has become a generalized epidemic and diagnosis rates are considerably higher among Indigenous peoples compared to non-Indigenous Canadians [3]. Indigenous peoples make up 4.3 % of the Canadian population yet accounted for 12.2 % of new HIV infections and 18.8 % of reported AIDS cases in 2011 [18]. In Australia, HIV diagnoses are higher among Aboriginal and Torres Strait Islander females than among non-Indigenous females even though the overall rate of HIV diagnoses in the Aboriginal and Torres Strait Islander population has remained stable [19]. In New Zealand, Maori men are more likely to test late for HIV than non-Maori men, leading to poorer health outcomes [20]. Although rates of HIV among Indigenous peoples in the United States, Australia and New Zealand are not substantially higher than among non-Indigenous peoples in those countries, disadvantageous social determinants place these populations at risk of future HIV generalised epidemics as seen in Canada.

Given this context, it is important to understand the determinants of HIV-related behaviors in these countries. With this in mind, we conducted a comprehensive literature review to assess the state of HIV knowledge and evidence generated over the past 30 years focusing on HIV-related behaviors and determinants.

## Methods

### Social Determinants of Health Framework

The Social Determinants of Health guide us to examine factors that influence health status from beyond the narrow remit of the health sector only and include poverty, inequality, history, social cohesion, marginalization and other issues. Our review of the literature and subsequent analysis has been guided by this thinking and the vast literature on social determinants [7, 24].

### Search Strategy and Information Sources

We followed by the ‘Preferred reporting items for systematic reviews and meta-analyses’ (PRISMA) guidelines [25] in the preparation of this literature review. In December 2013, author JN conducted searches in Medline, Embase and Web of Science. Search terms included HIV and AIDS as well as the four countries of focus: Australia, Canada, New Zealand and the United States. We examined the methods sections of systematic reviews on Indigenous peoples to be as inclusive as possible with the search terms. The search terms for Indigenous peoples included:

((American Native Continental Ancestry Group) OR (Oceanic Ancestry Group) OR (Maori) OR (Native Americ*) OR (Indigeno*) OR (First Nation*) OR (aborigin*) OR (torres strait*) OR (inuit) OR (native hawai*) OR (Alaska* native*) OR (metis)).

### Eligibility Criteria

Inclusion criteria included: (a) the paper was available in English, (b) the paper focused on HIV or a recognized determinant and/or behavior directly relevant to HIV transmission (e.g. injecting drug use, sex work) and, (c) the paper focused on Indigenous peoples. There was no date of publication limit. Papers that only mentioned Indigenous peoples or that had Indigenous peoples as part of a larger sample but did not analyze the data by Indigenous status specifically were excluded. Papers that only provided information on basic epidemiology were also excluded.

Titles were reviewed by three authors (JN, TG, CA) with inclusion of any article that was deemed relevant by any one of the three authors based on inclusion of a clear determinant and/or behavior. Then the same three authors reviewed abstracts for those articles to further narrow the list. Full-text articles were then retrieved and information extracted and reviewed. Where there was indecision, the three authors discussed each article to ascertain the final inclusion list. JN and TG then reviewed reference lists of relevant articles for possible additional material.

### Data Extraction and Synthesis

Using the final list, information was extracted by author TG from each study on country of focus, population group(s), year of publication, methods employed, sample size, and key findings. A tailored data extraction form was used. Each full article was read by multiple authors. We reviewed various HIV and behavioral frameworks to determine which suited the study best based on what themes were emerging while being guided by the social determinants documentation. Meade and Sikkema’s theoretical model of HIV risk behavior among adults with mental illness captured a number of the key themes, behaviors and determinants found through our synthesis [26]. Using Karina Walters’ term of “indigenization” [27], we “indigenized” Meade and Sikkehma’s framework to reflect our findings through an iterative process.

## Results

**Fig. 1:**
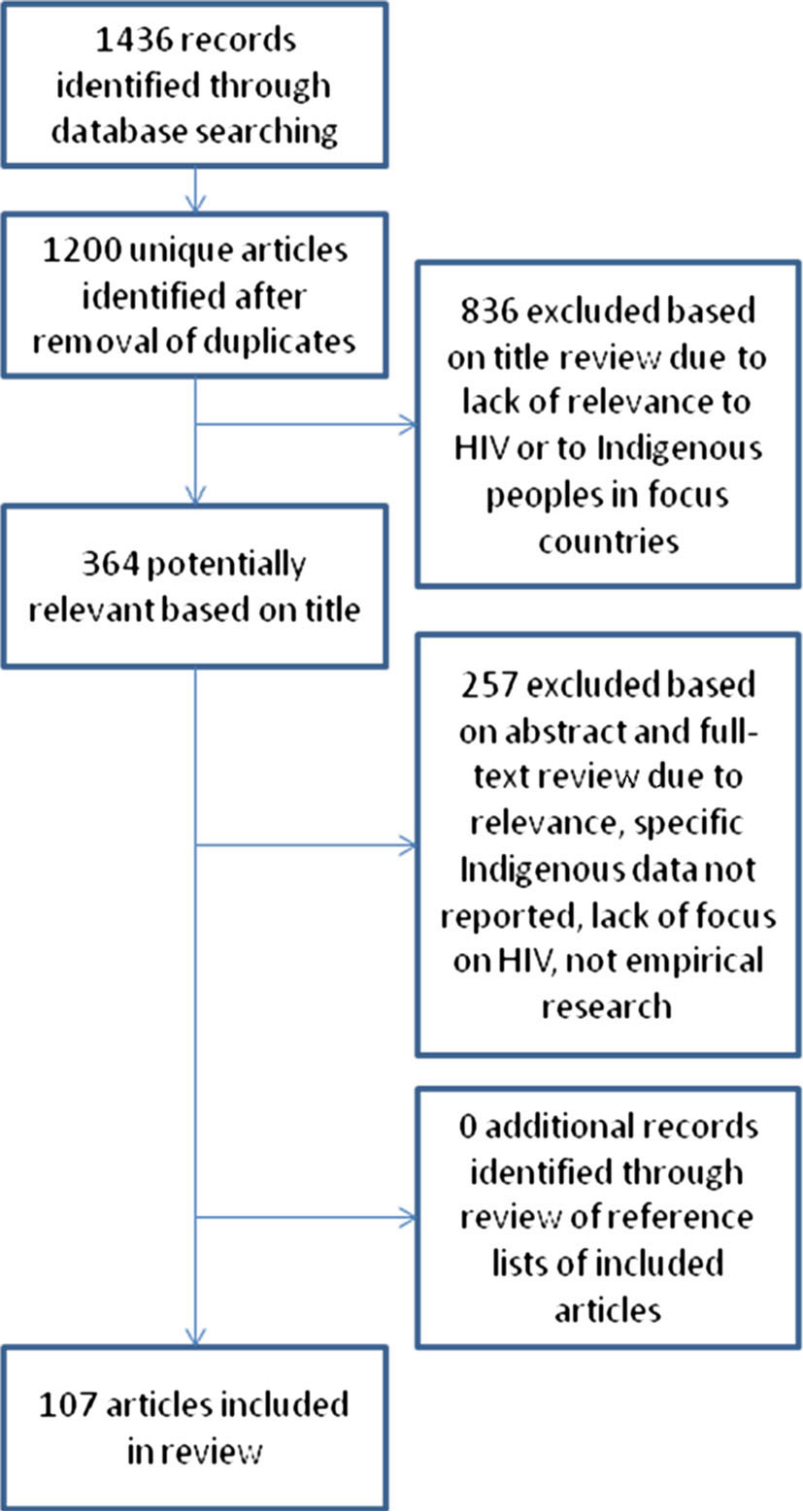
Selection of articles for comprehensive literature review of HIV and Indigenous populations

Through the search strategy, 1200 articles were identified. After a series of reviews of titles, abstracts and full texts, 107 studies were included in this review (Fig. 1) which are all identified in Table 2.

**Table 2:** Identified articles on HIV-related behaviors and determinants among Indigenous peoples

First author	Date	Title	Country	Focus group
Neilsen, G [29]	1993	Human immunodeficiency virus notifications for aborigines and Torres strait islanders in Queensland	Australia	Population-wide
Roberts, K [111]	1997	Condom use in a group of Aboriginal women	Australia	Women
Miller, P [52]	1998	Private business: the uptake of confidential HIV testing in remote Aboriginal communities on the Anangu Pitjantjatjara Lands	Australia	Population-wide
Guthrie, J [112]	2000	HIV and AIDS in Aboriginal and Torres Strait Islander Australians: 1992–1998. The National HIV Surveillance Committee	Australia	Population-wide
Wright, M [113]	2005	Fulfilling prophecy? Sexually transmitted infections and HIV in Indigenous people in Western Australia	Australia	Population-wide
Lawrence, C [78]	2006	Risk behaviour among Aboriginal and Torres Strait Islander gay men: comparisons with other gay men in Australia	Australia	MSM
Gilles, M [47]	2007	Perinatal HIV transmission and pregnancy outcomes in Indigenous women in Western Australia	Australia	PLWH
Newman, C [48]	2007	Everything is okay’: The influence of neoliberal discourse on the reported experiences of Aboriginal people in Western Australia who are HIV-positive	Australia	Population-wide
Newman, C [49]	2007	Barriers and incentives to HIV treatment uptake among Aboriginal people in Western Australia	Australia	PLWH
Thompson, S [50]	2009	Slowed right down: Insights into the use of alcohol from research with Aboriginal Australians living with HIV	Australia	PLWH
Fagan, P [51]	2010	Knowledge, attitudes and behaviours in relation to safe sex, sexually transmitted infections (STI) and HIV/AIDS among remote living north Queensland youth	Australia	Youth
Bryant, J [55]	2011	Safer sex and condom use: a convenience sample of Aboriginal young people in New South Wales	Australia	Youth
Lea, T [80]	2013	Elevated reporting of unprotected anal intercourse and injecting drug use but no difference in HIV prevalence among Indigenous Australian men who have sex with men compared with their Anglo-Australian peers	Australia	MSM
Paquette, D [84]	2013	Risk Practices Among Aboriginal People Who Inject Drugs in New South Wales, Australia	Australia	PWID
Brassard, P [62]	1996	Needs assessment for an urban native HIV and AIDS prevention program	Canada	Population-wide
Mill, J [66]	1997	HIV risk behaviors become survival techniques for Aboriginal women	Canada	Women
Myers, T [114]	1997	Differences in sexual risk-taking behavior with state of inebriation in an Aboriginal population in Ontario, Canada	Canada	Population-wide
Calzavara, L [115]	1998	Condom use among Aboriginal people in Ontario, Canada	Canada	Population-wide
Calzavara, L [63]	1999	Sexual partnering and risk of HIV/STD among Aboriginals	Canada	Population-wide
Heath, K [68]	1999	HIV-associated risk factors among young Canadian Aboriginal and non-Aboriginal men who have sex with men	Canada	MSM
Mill, J [116]	2000	Describing an explanatory model of HIV illness among Aboriginal women	Canada	PLWH
Martin, J [117]	2002	HIV and hepatitis B surveillance in first nations alcohol and drug treatment centres in British Columbia, Canada, 1992–2000	Canada	PWID
Craib, K [118]	2003	Risk factors for elevated HIV incidence among Aboriginal injection drug users in Vancouver	Canada	PWID
Mitchell, C [93]	2004	Identifying diverse HIV risk groups among American Indian young adults: the utility of cluster analysis	Canada	Youth
Hogg, R [79]	2005	HIV prevalence among Aboriginal British Columbians	Canada	Population-wide
Bucharski, D [65]	2006	You need to know where we’re coming from: Canadian Aboriginal women’s perspectives on culturally appropriate HIV counseling and testing	Canada	Women
Lima, V [119]	2006	Aboriginal status is a prognostic factor for mortality among antiretroviral naive HIV-positive individuals first initiating HAART	Canada	PLWH
Miller, C [31]	2006	Elevated rates of HIV infection among young Aboriginal injection drug users in a Canadian setting	Canada	Youth, PWID
Miller, C [85]	2006	Inadequacies in antiretroviral therapy use among Aboriginal and other Canadian populations	Canada	Population-wide
Wardman, D [120]	2006	HIV/AIDS: Testing and risk behaviors among British Columbia’s rural Aboriginal population	Canada	Population-wide
Wood, E [33]	2006	Slower uptake of HIV antiretroviral therapy among Aboriginal injection drug users	Canada	PWID, PLWH
Callaghan, R [121]	2007	Mobility patterns of Aboriginal injection drug users between on- and off-reserve settings in Northern British Columbia, Canada	Canada	PWID
Larkin, J [69]	2007	HIV risk, systemic inequities, and Aboriginal youth: widening the circle for HIV prevention programming	Canada	Youth
Shannon, K [122]	2007	Sexual and drug-related vulnerabilities for HIV infection among women engaged in survival sex work in Vancouver, Canada	Canada	Women
Spittal, P [32]	2007	The Cedar Project: Prevalence and correlates of HIV infection among young Aboriginal people who use drugs in two Canadian cities	Canada	Youth, PWID
Barlow, K [94]	2008	Culturally Competent Service Provision Issues Experienced By Aboriginal People Living With HIV/AIDS	Canada	PLWH
Marshall, B [70]	2008	High prevalence of HIV infection among homeless and street-involved Aboriginal youth in a Canadian setting	Canada	Youth
Mehrabadi, A [30]	2008	The Cedar Project: A comparison of HIV-related vulnerabilities amongst young Aboriginal women surviving drug use and sex work in two Canadian cities	Canada	Youth, PWID, Women
Mehrabadi, A [72]	2008	Gender differences in HIV and Hepatitis C related vulnerabilities among Aboriginal young people who use street drugs in two Canadian cities	Canada	Youth, PWID, Women
Mill, J [56]	2008	HIV testing and care in Canadian Aboriginal youth: a community based mixed methods study	Canada	Youth
Wood, E [64]	2008	Burden of HIV infection among Aboriginal injection drug users in Vancouver, British Columbia	Canada	Youth, PWID
McCall, J [96]	2009	Struggling to survive: the difficult reality of Aboriginal women living with HIV/AIDS	Canada	PLWH
Orchard, T [123]	2010	Factors behind HIV testing practices among Canadian Aboriginal peoples living off-reserve	Canada	Population-wide
Shaw, S [124]	2010	Increased risk for hepatitis C and HIV associated with solvent use among Canadian Aboriginal injection drug users	Canada	PWID
Worthington, C [110]	2010	HIV testing experiences of Aboriginal youth in Canada: service implications	Canada	Youth
Devries, K [71]	2011	Boyfriends and booty calls: sexual partnership patterns among Canadian Aboriginal young people	Canada	Youth
Duncan, K [125]	2011	HIV incidence and prevalence among Aboriginal peoples in Canada	Canada	Population-wide
Jaworsky, D [99]	2011	Comparison of late HIV diagnosis as a marker of care for Aboriginal versus non-Aboriginal people living with HIV in Ontario	Canada	PLWH
Martin, L [53]	2011	All-cause and HIV-related mortality rates among HIV-infected patients After initiating highly active antiretroviral therapy: the impact of Aboriginal ethnicity and injection drug use	Canada	PWID
Monette, L [97]	2011	Inequalities in determinants of health among Aboriginal and Caucasian persons living with HIV/AIDS in Ontario: results from the positive spaces, healthy places study	Canada	PLWH
Brondani, M [106]	2012	Community-based research among marginalized HIV populations: issues of support, resources, and empowerment	Canada	PLWH
Lemstra, M [126]	2012	Risk indicators associated with injection drug use in the Aboriginal population	Canada	PWID, Women
Cain, R [73]	2013	The experience of HIV diagnosis among Aboriginal people living with HIV/AIDS and depression	Canada	PLWH
Chavoshi, N [74]	2013	The cedar project: understanding barriers to consistent condom use over time in a cohort of young Indigenous people who use drugs	Canada	PWID
Gunther, M [127]	2013	Treatment beliefs, illness perceptions, and non-adherence to antiretroviral therapy in an ethnically diverse patient population	Canada	PLWH
Siemieniuk, R [76]	2013	Prevalence, clinical associations, and impact of intimate partner violence among HIV-infected gay and bisexual men: a population-based study	Canada	MSM
Martin, L [128]	2010	Rates of initial virological suppression and subsequent virological failure after initiating highly active antiretroviral therapy: the impact of Aboriginal ethnicity and injection drug use	Canada	Population-wide
Grierson, J [98]	2004	Mate Aaraikore A Muri Ake Nei: experiences of Maori New Zealanders living with HIV	New Zealand	PLWH
Dickson, N [20]	2012	Late presentation of HIV infection among adults in New Zealand: 2005-2010	New Zealand	Population-wide
Hall, R [28]	1990	Assessment of AIDS knowledge, attitudes, behaviors, and risk level of Northwestern American Indians	USA	Population-wide
Metler, R [129]	1991	AIDS surveillance among American Indians and Alaska natives	USA	Population-wide
Conway, G [61]	1992	HIV infection in American Indians and Alaska natives: surveys in the Indian health service	USA	Population-wide
Lieb, L [36]	1992	Racial misclassification of American Indians with AIDS in Los Angeles County	USA	PLWH
No Author [130]	1998	HIV/AIDS among American Indians and Alaskan Natives–United States, 1981–1997	USA	PLWH
Fenaughty, A [45]	1998	Sex partners of Alaskan drug users: HIV transmission between white men and Alaska Native women	USA	PWID, Women
Fenaughty, A [82]	1998	Sex partners of native American drug users	USA	PWID, Women
Baldwin, J 44]	1999	HIV/AIDS risks among native American drug users: key findings from focus group interviews and implications for intervention strategies	USA	Population-wide
Baldwin, J [131]	2000	Alcohol as a risk factor for HIV transmission among American Indian and Alaska native drug users	USA	PWID
Bouey, P [95]	2000	The Ahalaya case-management program for HIV-infected American Indians, Alaska Natives, and Native Hawaiians: quantitative and qualitative evaluation of impacts	USA	PLWH
Fisher, D [46]	2000	Alaska native drug users and sexually transmitted disease: results of a five-year study	USA	PWID, Women
Reynolds, G [83]	2000	Unemployment, drug use, and HIV risk among American Indian and Alaska native drug users	USA	PWID
Stevens, S [132]	2000	HIV drug and sex risk behaviors among American Indian and Alaska Native drug users: gender and site differences	USA	Population-wide
Walters, K [39]	2000	Patterns and predictors of HIV risk among urban American Indians	USA	Population-wide
Diamond, C [34]	2001	HIV-infected American Indians/Alaska natives in the Western United States	USA	PLWH
Ka’opua, L [41]	2001	Treatment adherence to an antiretroviral regime: the lived experience of Native Hawaiians and kokua	USA	PLWH
Morrison-Beedy, D [90]	2001	HIV risk behavior and psychological correlates among native American women: an exploratory investigation	USA	Women
Odo, C [43]	2001	Eo na Mahu o Hawai’i: the extraordinary health needs of Hawai’i’s Mahu	USA	Women
Hobfoll, S [67]	2002	The impact of perceived child physical and sexual abuse history on Native American women’s psychological well-being and AIDS risk	USA	Women
Mitchell, C [57]	2002	Structure of HIV knowledge, attitudes, and behaviors among American Indian young adults	USA	Youth
Ramirez, J [92]	2002	Effects of fatalism and family communication on HIV/AIDS awareness variations in native American and Anglo parents and children	USA	Youth
Denny, C [133]	2003	Surveillance for health behaviors of American Indians and Alaska Natives. Findings from the behavioral risk factor surveillance system, 1997–2000	USA	Population-wide
Mueller, C [134]	2003	Psychosocial adjustment of Native Hawaiian women living with HIV/AIDS: the central role of affective bonds	USA	PLWH
Ashman, J [135]	2004	Health and support service utilization patterns of American Indians and Alaska Natives diagnosed with HIV/AIDS	USA	PLWH
Bertolli, J [136]	2004	Surveillance systems monitoring HIV/AIDS and HIV risk behaviors among American Indians and Alaska natives	USA	Population-wide
Ka’opua, L [41]	2004	Treatment adherence among Native Hawaiians living with HIV	USA	PLWH
Foley, K [137]	2005	Using motivational interviewing to promote HIV testing at an American Indian substance abuse treatment facility	USA	PWID
Gilley, B [138]	2005	Cultural investment: providing opportunities to reduce risky behavior among gay American Indian males	USA	MSM
McNaghten, A [139]	2005	Epidemiologic profile of HIV and AIDS among American Indians/Alaska natives in the USA through 2000	USA	Population-wide
Nebelkopf, E [37]	2005	Holistic native network: integrated HIV/AIDS, substance abuse, and mental health services for native Americans in San Francisco	USA	PLWH
Saylors, K [75]	2005	Native women, violence, substance abuse and HIV risk	USA	Women
Gorgos, L [101]	2006	Determinants of survival for Native American adults with HIV infection	USA	PLWH
Lapidus, J [88]	2006	HIV-related risk behaviors, perceptions of risk, HIV testing, and exposure to prevention messages and methods among urban American Indians and Alaska Natives	USA	Population-wide
Marsiglia, F [108]	2006	HIV/AIDS protective factors among urban American Indian youths	USA	Youth
Simoni, J [38]	2006	Victimization, substance use, and HIV risk behaviors among gay/bisexual/two-spirit and heterosexual American Indian men in New York City	USA	MSM
Gilley, B [86]	2007	Linking ‘white oppression’ and HIV/AIDS in American Indian etiology: conspiracy beliefs among MSMs and their peers	USA	MSM
Johnson, J [35]	2007	HIV/AIDS, substance abuse, and hepatitis prevention needs of Native Americans living in Baltimore: In their own words	USA	Population-wide
Kaufman, C [58]	2007	Culture, context, and sexual risk among Northern Plains American Indian Youth	USA	Youth
Vernon, I [140]	2007	American Indian women, HIV/AIDS, and health disparity	USA	Women
Ellingson, L [40]	2008	HIV risk behaviors among mahuwahine (Native Hawaiian Transgender Women)	USA	Women
Lowe, J [109]	2008	A cultural approach to conducting HIV/AIDS and hepatitis C virus education among native American adolescents	USA	Youth
Cassels, S [81]	2010	Sexual partner concurrency and sexual risk among gay, lesbian, bisexual, and transgender American Indian/Alaska natives	USA	MSM
Iralu, J [100]	2010	Risk Factors for HIV disease progression in a rural Southwest American Indian population	USA	PLWH
Burks, D [77]	2011	American Indian gay, bisexual and two-spirit men: a rapid assessment of HIV/AIDS risk factors, barriers to prevention and culturally-sensitive intervention	USA	MSM
Nelson, K [141]	2011	‘I’ve had unsafe sex so many times why bother being safe now?’: the role of cognitions in sexual risk among American Indian/Alaska native men who have sex with men	USA	MSM
Sondag, K [87]	2011	HIV/AIDS among American Indians/Alaska natives living in Montana: a descriptive study	USA	Population-wide
Leston, J [91]	2012	Alaska native and rural youth views of sexual health: a focus group project on sexually transmitted diseases, HIV/AIDS, and unplanned pregnancy	USA	Youth
Anastario, M [60]	2013	Sexual risk behavior and symptoms of historical loss in American Indian men	USA	Population-wide
Pearson, C [142]	2013	A cautionary tale: risk reduction strategies among urban American Indian/Alaska native men who have sex with men	USA	MSM

*MSM* men who have sex with men, *PLWH* people living with HIV, *PWID* people who inject drugs

**Fig. 2:**
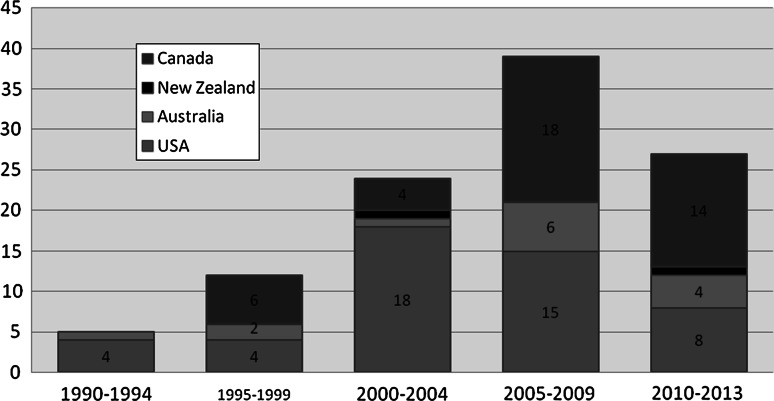
Number of articles published by year and focus country

By country, 49 of the 107 (45.8 %) articles were based on American data followed by 42 (39.3 %) from Canada, 14 (13.1 %) from Australia and two (1.9 %) from New Zealand (Fig. 2). The review identified articles concerning HIV among Indigenous peoples dating back more than 20 years (Table 3). For instance, we discovered a 1990 study, which concluded that American Indians in the Pacific Northwest of the USA were considered more ‘vulnerable’ to HIV than the general population [28]. The first article on rates of HIV infection in Australia’s Aboriginal and Torres strait islander population was published in 1993 [29]. In general, the number of relevant articles has been increasing over time with a peak in the 2005–2009 period.

**Table 3:** Number of articles by group and country

	Australia	Canada	New Zealand	United States
Population-wide	5	9	1	13
PLWH	3	11	1	11
MSM	2	2		7
Transgender				2
PWID	1	14		6
Youth	2	11		6
Women	1	6		7

Total is more than the number of included articles as categories were not mutually exclusive

*PLWH* people living with HIV, *MSM* men who have sex with men, *PWID* people who inject drugs

Overall, 77 (72 %) of the articles used quantitative methods including cross-sectional studies, analysis of medical records, use of surveillance data and retrospective analyses; 27 (25 %) used qualitative methods including focus group and face to face interviews; and 4 (4 %) used both qualitative and quantitative methods. Sample sizes ranged widely in both types of studies. Of the 26 studies examining PLWH, 10 (38 %) used qualitative methods compared to only one of 21 (5 %) studies focused on PWID (Table 4). Australian studies were the most likely to use qualitative methods.

**Table 4:** Article methodology by group and country

Group/Country	Quantitative	Qualitative	Mixed Methods
Australia	9	5	
Canada	30	11	1
New Zealand	2		
United States	36	11	2
Population-wide	22	5	1
PLWH	16	10	
MSM	6	3	
Transgender	2		
PWID	20	1	
Youth	13	4	2
Women	10	4	

Total is more than the number of included articles as categories were not mutually exclusive. Mixed Methods represents studies that included both quantitative and qualitative components

*PLWH* people living with HIV, *MSM* men who have sex with men, *PWID* people who inject drugs

### Demographics

The groups of Indigenous peoples upon which the identified articles focused varied considerably, with some concentrating on people living with HIV (PLWH) and others on youth or women. Table 3 reveals that 21 studies focused on PWID, 14 on women and 13 on MSM and transgender people.

Canadian studies had a particular focus on groups identified as ‘vulnerable’ and behaviors defined as ‘high-risk’, with 14 articles on PWID and 11 on youth—with a locus of study activity in and around Vancouver, British Columbia [30–33]. The American articles featured a mix of urban-based studies [34–39] as well as those conducted in Hawaii [40–43] and Alaska [44–46]. Australian articles included a number from Western Australia [47–50] and a handful from remote communities [51, 52].

Overall, articles tended to focus on a generalised Indigenous population and typically identified characteristics of HIV transmission and ‘high-risk’ behavior. Articles revealed higher rates of injecting drug use and heterosexual contact as well as social disadvantage, higher incidence of sexually-transmitted infections (STIs) and poorer access to health services [19, 35, 53] among Indigenous peoples compared to non-Indigenous people [54].

Inconsistent and low condom use represents an immediate risk factor for HIV infection, which also leads to high rates of STIs in Indigenous populations [55–58]. One study from the North American Arctic [59] as well as one study focused on Native Americans and Native Alaskans [17] found higher rates of STIs as well as lower rates of condom use among those Indigenous groups. An American study revealed that American Indian men living in Montana who experienced greater symptoms of “historical loss” had an increased likelihood of having multiple concurrent sex partners [60].

Similar rates of HIV were identified among urban and rural Indigenous peoples despite the suggestion that those in urban areas might be exposed to conditions which are more conducive to infection [61, 62]. Indeed, Calzavara and colleagues found that almost 50 % of Indigenous peoples living on reserve in Ontario had sexual partners from outside the community—suggesting that remote communities are not insulated from transmission of HIV [63].

In some of the review countries, Indigenous youth are overrepresented in reported HIV and AIDS cases among youth [2, 64]. In addition, two articles focused on Native Hawaiian Transgender people, highlighting behaviors and determinants—including sex work, illegal drugs, domestic violence and lack of health insurance—which create greater exposure to HIV infection than observed in other Native Hawaiians [40, 43].

### Determinants and Behaviors

**Fig. 3:**
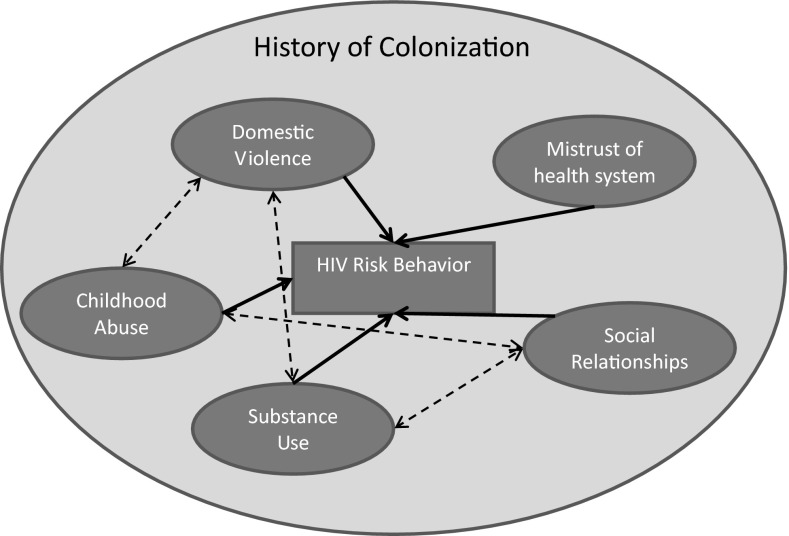
Determinants of HIV Risk Behavior among Indigenous Peoples (adapted from Meade and Sikkema [26]) *Solid lines* represent determinants that impact HIV behavior. Note: *Dashed lines* represent hypothesized relationships between determinants

Based on review of the 107 articles, determinants of HIV risk were organized into the following themes based on an adaptation of Meade and Sikkema’s HIV risk model [26] and using the social determinants framework as guidance: substance use, childhood abuse, domestic violence, social relationships, and mistrust of health services (Fig. 3). These are all underpinned by a history of colonization.

### Childhood Abuse

Childhood abuse—both sexual and emotional—was highlighted in a number of articles as a contributor to future behavior that increased exposure to HIV infection. Two Canadian qualitative studies with Indigenous women found that repeated childhood abuse was experienced by the majority of participants [65, 66], beginning as early as 4 years of age and often leading to alcohol and drug abuse. Similarly, a US study found that Indigenous women who were physically and emotionally abused as children had 5.14 times greater odds of having a STI in their lifetime than did women who experienced only marginal or no physical-emotional abuse [67]. A Vancouver-based study compared Indigenous and non-Indigenous MSM and found that Indigenous respondents were more likely to report sexual abuse during childhood [68].

Foster care among Indigenous youth has been linked to poor emotional health, increased drug use and greater involvement in sex work [69]. Indigenous youth are also more likely than non-Indigenous youth to experience physical and emotional trauma (often intergenerational), which increases their exposure to sexual exploitation and drug use. Similarly, family instability and sexual abuse at a young age have been associated with HIV positive status among Indigenous youth [30, 70, 71]. More broadly, early sexual initiation, high-risk partnership patterns and involvement in sex work increase Indigenous youth’s HIV risk [71, 72]. A qualitative study from Canada revealed that the “legacies of residential schools… [and] the disruption of traditional culture” shaped how Indigenous individuals experienced their HIV diagnosis [73].

### Domestic Violence

One Canadian study found that inconsistent condom use by young women was predicted by having experienced recent sexual abuse [74]. A US study revealed that almost two-thirds of the participating Indigenous women had experienced coercive sexual contact [75]. These women attributed some of the challenges they faced to the breakdown of Indigenous support structures and traditional teachings.

Domestic violence was not only found among Indigenous women. Research from Alberta, Canada revealed that HIV-positive Indigenous gay and bisexual men were 2.48 times more likely to report intimate partner violence than white counterparts [76].

### Social Relationships

Eleven articles focused specifically on Indigenous MSM and a number of these identified social disadvantage, including high rates of unemployment, low educational achievement, poor access to health resources, especially condoms, and poor access to health services as significant barriers to HIV prevention [68, 77–79]. Two Australian studies revealed that Indigenous respondents were significantly more likely to have engaged in unprotected anal intercourse with casual partners than non-Indigenous MSM (23.5 vs. 20.7 %; p = 0.01) [78, 80]. A multi-centre US study among urban Indigenous MSM found a high rate of HIV prevalence (34 %) among those who only had sex with men [81].

Two articles included specific reference to two-spirit individuals [38, 77]; two-spirit is a concept of Indigenous gender identity—and not specifically of sexual orientation—whereby individuals have a blend of both male and female spirits. Victimization and discrimination were identified as putting Indigenous MSM or two-spirit men at higher risk of HIV infection [38]. Once again, mistrust of health service providers was identified as a significant barrier to HIV prevention, testing and treatment [77].

### Substance Use

The majority of articles focusing on PWID were from Alaska and Canada, with British Columbia featuring prominently. In fact, only one study on Indigenous PWID originated in Australia. However, across the four countries, injecting drug use was commonly reported as a form of pain relief or alleviation of past trauma and abuse and was often associated with sex work. [45, 72, 82].

Compared to non-Indigenous PWID, Indigenous PWID demonstrated more frequent use, a higher likelihood of sharing equipment, lower levels of accessing risk reduction programs such as methadone clinics or needle-exchange programs, as well as a history of incarceration [31, 83, 84]. With regard to treatment, Indigenous PWID appear to take longer to access anti-retroviral treatment and display worse response rates to therapy than non-Indigenous PWID [33, 53].

Substance use, particularly injection drug use is strongly associated with HIV infection among Indigenous youth in Canada, where they are often overrepresented among youth who inject drugs in large urban centres [30, 32, 72, 85]. An Australian study of 1208 Indigenous MSM in urban areas found that participants were significantly more likely than non-Indigenous MSM to have injected drugs in the 6 months prior (10.8 vs. 5.9 %; p < 0.001) [78].

### Mistrust of Health System

Mistrust of health services was common [48] with one article from 2007 reporting that a third of respondents believed that ‘white’ people intentionally spread HIV among native peoples [86]. Two papers discussed barriers to testing for Indigenous peoples, with stigma and lack of confidentiality being identified as significant impediments [35, 87]. The consequent late testing for HIV was highlighted as a contributing factor to poor health outcomes for Indigenous peoples [88].

In two of the studies focused particularly among women, women expressed distrust of the health system as well as fear of judgment and discrimination by health providers [65, 89]. One study suggested that this mistrust cascaded into misperceptions of personal risk: one survey on HIV risk behavior and perceptions among Native American women in the US revealed that those who were classified as higher risk actually felt less vulnerable to HIV and were less ready to change their behaviors compared with those classified as lower-risk [90].

Likewise, the denial of HIV within Indigenous communities was cited as a critical determinant of risk for Indigenous youth. As a result, although Indigenous youth experience greater actual risk of contracting HIV, awareness and perception of that risk are generally low [51, 56, 91, 92]. Moreover, Indigenous youth and their parents report low levels of knowledge about HIV, which often leads to low perceived vulnerability, diminished prevention and reduced testing [51, 93].

Several articles highlighted the need for greater cultural sensitivity and competencies among health staff, care providers and health programs serving Indigenous communities [37, 49, 94, 95]. Discrimination and judgmental behavior on the part of health care providers were frequently reported as a deterrent to seeking medical treatment [96–98].

While the review did not identify differences between Indigenous and non-Indigenous peoples’ physiological responses to treatment, low income, high rates of unemployment, younger age, being female and having experienced incarceration or homelessness were found to contribute to poor treatment outcomes among Indigenous peoples [34, 97, 99, 100]. The two most significant factors affecting treatment adherence and outcomes among Indigenous peoples were ongoing substance abuse and the timing of treatment uptake [33, 41, 50, 101].

## Discussion

This review of literature reveals a long history of studies that identify the threat of HIV infection among Indigenous peoples in Australia, Canada, New Zealand and the United States. More than 100 articles on HIV-related behaviors and determinants have been published since 1990, highlighting various Indigenous groups including MSM, PWID and youth that demonstrate the impact of areas such as childhood abuse, domestic violence and substance use on HIV behavior.

A quarter of studies used qualitative methods and most of those examined perceptions and social aspects of HIV among Indigenous peoples through face to face interviews. These studies were able to extract some of the particular challenges that Indigenous peoples face in addressing HIV. The quantitative studies published in the early years of the epidemic often relied on surveillance data but more recent studies have utilized specific studies designed for Indigenous populations.

Factors identified as contributing to HIV risk include social disadvantage, poor access to health services, high rates of injecting drug use and STIs, as well as exposure to stigma and discrimination. It is worth noting that there was little differentiation between remote and urban-dwelling Indigenous peoples in both Australia and Canada, with people in both locations being exposed to similar levels of disadvantage. Many of the determinants associated with HIV risk also contribute to mistrust of health services, which in turn contributes to late testing for HIV among Indigenous peoples and subsequent poor health outcomes.

The review revealed a number of themes associated with HIV risk for particular population groups. For instance, childhood sexual abuse was experienced by substantial numbers of HIV-positive Indigenous women and MSM, who also experience considerable stigma and poor access to services. A number of studies also identified injecting drug use as a significant determinant of HIV risk among Indigenous populations.

Although we included articles that explicitly focused on Indigenous peoples, we acknowledge that Indigenous peoples do not always self-identify as ‘Indigenous’ so that under-reporting or mis-reporting of indigeneity can occur. Some articles might have been missed due to our inclusion criteria, which required some substantial focus on Indigenous peoples and the emphasis on behavior did exclude articles that focused only on epidemiology. We also acknowledge that some of the early HIV publications were in grey literature and therefore were beyond the scope of this review. Overall however, other reviews have acknowledged substantial data gaps with regard to Indigenous health [102].

## Conclusion

This literature review of Indigenous HIV research demonstrates—in a number of the articles examined—a persistent focus on vulnerability and risk, which appears to have driven Indigenous HIV research since the early days of the epidemic. As international strategies focus on treatment access, greater effort is needed to address the broader determinants of risk among Indigenous peoples as well as developing approaches that focus on empowerment to ensure inclusion of Indigenous peoples. Consideration should be given to the strength and resilience of Indigenous peoples [103, 104] that promote spiritual wellness while living with HIV and peer Indigenous leadership that show the importance of collective versus individual perspectives to HIV prevention and treatment. In order to reduce HIV disparities and enhance the health and well-being of Indigenous peoples affected by HIV, it is essential that we find viable alternatives to past strategies to overcoming HIV in Indigenous communities.

Indigenous peoples have a long history of mistrust of health and social services, which has been an ongoing feature of colonization and elements of which continue to this day [105]. Therefore, involvement of Indigenous peoples in HIV prevention and treatment programs is essential. Baldwin and colleagues strongly recommended directly involving members of Indigenous communities in conducting interventions and utilizing tribally-relevant forms of communication to deliver messages [44]. Articles also highlight a desire for health services delivered by Indigenous people for Indigenous people [106] and the importance of health workers understanding the trauma and history of Indigenous peoples in order to provide appropriate care [107]. Protective factors against HIV infection have been identified as having positive relationships and open communication with their families [108], the integration of cultural values and beliefs into HIV prevention programs [109] as well as supportive HIV education and testing environments [110].

A greater focus on the strengths of Indigenous communities needs to be complemented by a leadership that promotes and supports the notion that HIV affects everyone within Indigenous communities—not just those living with the virus. By improving access to HIV services, providing comprehensive prevention information as well as resources, significant headway will be made in stemming the impact of HIV on Indigenous communities throughout the world.
